# Wide-Range, Low-Hysteresis Soft Sensor with Architecture-Inspired Design Enabled by Femtosecond Laser-Induced Self-Growth

**DOI:** 10.3390/s26092784

**Published:** 2026-04-29

**Authors:** Ziyue Yu, Changhao Ji, Xinyue Gao, Yu Li, Cheng Yang, Fawei Guo, Jianglin Fu, Yin Feng, Hongxuan Zhao, Yu Long

**Affiliations:** 1 State Key Laboratory of Featured Metal Materials and Life-Cycle Safety for Composite Structures, Guangxi University, Nanning 530004, China; 18240438586@163.com (Z.Y.);; 2 Institute of Laser Intelligent Manufacturing and Precision Processing, School of Mechanical Engineering, Guangxi University, Nanning 530004, China; 3 School of Mechanical Engineering, Beijing Institute of Technology, Beijing 100089, China

**Keywords:** femtosecond laser microengineering, architecture-inspired design, low hysteresis, wide working range, flexible pressure sensor

## Abstract

Resolving the dichotomy between wide detection ranges and low mechanical hysteresis remains a critical challenge in flexible electronics, largely governed by the intrinsic viscoelastic creep of polymeric dielectrics. Drawing inspiration from the distinctive load-bearing mechanisms of traditional Chinese *Sparrow Brace* architecture, we report a mechanically optimized tilted micro-architecture designed to enhance structural resilience. Unlike conventional soft elastomeric pillars that easily succumb to mechanical failure, this BOPS-based tilted geometry provides excellent load-bearing capacity, effectively preventing premature failure. Finite element analysis (FEA) confirms that this tilted geometry forces a fundamental shift from conventional bulk compression to structural bending. Because this bending-dominated architecture drives rapid elastic recovery, it significantly mitigates the severe effects of the polymer’s viscoelastic creep under the tested loading conditions, achieving reliable signal reversibility with low hysteresis. We fabricated this specific architecture via programmable femtosecond laser direct writing (FsLDW) on biaxially oriented polystyrene (BOPS) films, harnessing the material’s entropy-driven self-growth kinetics. By merging this localized growth mechanism with the architectural design, we effectively bypassed the complexities of traditional molding, achieving mask-free, in situ growth of large-scale, highly uniform dielectric micro-arrays. The resulting sensor delivers a remarkably broad working range (up to ~2.28 MPa) coupled with a negligible recovery error (~1.3%), an agile dynamic response (~70/80 ms), and consistent operational durability. Ultimately, this work combines architecture-inspired structural design with advanced femtosecond laser surface microengineering, providing a conceptually novel and scalable pathway for next-generation flexible sensing.

## 1. Introduction

Flexible capacitive pressure sensors are central to the burgeoning fields of wearable health monitoring, soft robotics, and human–machine interfaces, owing to their distinct advantages in conformability, lightweight architecture, and reliable signal readout [1,2,3]. To accommodate diverse applications from delicate physiological signal detection to vigorous human motion monitoring, it is highly desirable for these sensors to deliver a broad working range while maintaining signal stability [4,5]. Within flexible sensor design, reconciling an expansive detection range with minimal mechanical hysteresis remains a persistent challenge. Fundamentally, this constraint arises from the widespread reliance on conventional elastomers, whose polymer networks naturally undergo viscoelastic creep and relaxation [6,7,8]. During continuous mechanical cycling or under substantial loads, these inherent creep behaviors accumulate as macroscopic hysteresis [9,10,11], steadily degrading signal reversibility and introducing baseline drift. Mitigating this inherent viscoelastic creep via targeted structural design thus emerges as a far more practical route than altering the polymer’s intrinsic nature [12].

Structural engineering of the dielectric layer has proven instrumental in modulating mechanical behavior. Various micro-architectures, such as pyramids, domes, and micro-pillars, have been extensively explored to enhance sensing performance [13,14,15,16]. For instance, Wang et al. [17] integrated porous nanofibers with micro-pyramid arrays to maximize compressibility for low-pressure detection. Similarly, Luo et al. [18] utilized vertically aligned micro-pillars to enhance sensitivity. Yet, these soft designs inevitably suffer from rapid structural saturation, restricting their effective working range to a narrow window of typically less than 60 kPa. Collectively, these studies underscore that conventional vertically aligned microstructures primarily undergo bulk compression, a deformation mode strongly coupled with the material’s viscoelastic nature [19,20].

To overcome these intrinsic limitations and extend the working range, researchers have sought to reduce the contribution of the dielectric layer’s viscoelastic creep to the mechanical response by changing deformation modes [21,22], such as introducing slanted or tilted micro-pillars. While these designs offer unique mechanical advantages, extending their reliable working range without exacerbating hysteresis under heavy loads remains a significant challenge. Furthermore, translating such intricate, tilted concepts into physical reality is hindered by practical fabrication hurdles. Traditional strategies often rely on physical templates, such as fixed masks [23] or pre-designed molds [24] to define tilted geometries. While effective, the former limits geometric adaptability for rapid design iterations, while the latter involves risky demolding steps that frequently compromise the integrity of high-aspect-ratio features. Although emerging additive techniques like 3D printing provide template-free flexibility [25,26], they typically entail elaborate material preparation and struggle with residual resin clearance from dense interstices, as well as the structural collapse of unsupported overhangs, when fabricating intricate micro-architectures on flexible films.

In this context, FsLDW stands out as an advanced microfabrication technique. The ultrashort pulses and high peak intensities of FsLDW enable sub-micron energy deposition through nonlinear absorption, inherently minimizing thermal damage [27,28,29]. Beyond conventional ablation, utilizing FsLDW to induce localized deformations in shape-memory polymers (SMPs) provides an innovative route for engineering smart materials [30,31]. By exploiting laser–material interactions, it is feasible to trigger stress release, enabling the “self-growth” of 3D micro-architectures from a planar film [32,33,34]. Building on this unique capability, we integrate architecture-inspired structural mechanics with programmable laser manufacturing to resolve the range-hysteresis conflict. We utilized BOPS films, a cost-effective shape-memory polymer, as the active substrate. The pre-stored internal stress within BOPS, governed by entropy elasticity, is locally released by femtosecond laser irradiation, initiating the in situ self-growth of microarrays. Conceptually, our micro-architecture translates the inclined load-bearing mechanics of the traditional Chinese *Sparrow Brace* into a functional tilted design. By synergizing femtosecond laser-induced self-growth with this architecture-inspired design, the resulting micro-arrays exhibit excellent load-bearing capacity, effectively resisting mechanical failure under heavy loads. As validated by comparative FEA, this tilted design effectively transforms vertical pressure into bending deformation, suppressing hysteresis by driving rapid elastic recovery. Enabled by this mechanical transition, the fabricated sensor consistently achieves an expansive working range (up to ~2.28 MPa) alongside a negligible recovery error (~1.3%) and long-term durability. This work highlights the distinct advantage of laser-induced self-growth in tailoring dielectric layers, establishing a robust strategy to effectively mitigate the trade-off between sensing range and mechanical hysteresis.

## 2. Results and Discussion

### 2.1. Mechanism of Entropy-Driven Shape Recovery and Macroscopic Quantification of Stored Strain

**Figure 1:**
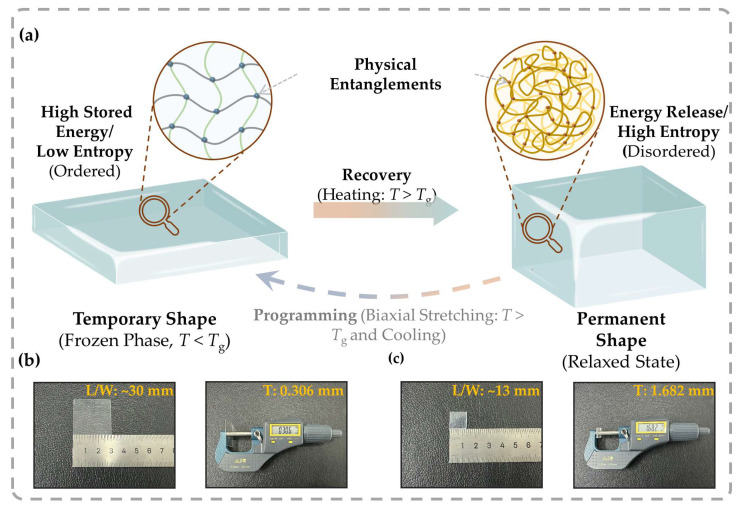
Thermodynamic mechanism and macroscopic verification of the shape memory effect in BOPS: (**a**) Schematic illustration of the entropy-driven recovery kinetics. The “temporary shape” (as-received BOPS) consists of kinetically frozen, highly oriented molecular chains (low entropy) stabilized by physical entanglements. Upon heating above the glass transition temperature (*Tg*), the stored elastic energy is released as chains recoil to a thermodynamically stable random-coil state (high entropy), driving the recovery to the “permanent shape.” (**b**,**c**) Macroscopic demonstration quantifying the deformation: (**b**) The initial BOPS thin sheet and (**c**) the recovered PS block after unrestricted thermal annealing. The comparison evidences a significant in-plane shrinkage accompanied by vertical thickening, confirming the release of stored strain.

The fabrication of self-grown micro-architectures is fundamentally underpinned by the entropy-driven shape-memory behavior of the BOPS substrate. Unlike chemically crosslinked shape-memory polymers, the “memory” in thermoplastic BOPS is governed by physical networks, arising primarily from chain entanglements and intermolecular forces [35,36], notably van der Waals interactions. Figure 1a illustrates this physical sequence. Through the pre-stretching process [37], polymer chains are forced into a highly oriented, low-entropy non-equilibrium state, which is then kinetically “frozen” below the glass transition temperature (*T_g_*). This process effectively locks high-density residual stresses within the glassy polymer matrix. Upon reheating above *T_g_*, the relaxation of physical constraints restores segmental mobility. Thermodynamically, the oriented chains spontaneously recoil into their high-entropy, random-coil equilibrium [35,36]. At the macroscopic level, this internal relaxation discharges the stored elastic energy, allowing the material to recover its original configuration.

To capture the deformation behavior guiding our structural design, we performed macroscopic unrestricted thermal annealing. Upon heating, the PS specimen exhibits pronounced anisotropic contraction (Figure 1b,c). This planar deformation is quantified by the linear shrinkage ratio (*S_L_*), defined as

(1) $$S_{L}=\frac{L_{0}-L}{L_{0}}\times 100\%$$

where *L*_0_ and *L* are the initial and final characteristic in-plane lengths, respectively. For our standard specimen (*L*_0_ = 30 mm, *L* ≈ 13 mm), this yields a nominal linear shrinkage of ~56.7%. Correspondingly, the film thickness increases from 0.306 mm to 1.682 mm. Although non-uniform edge effects and measurement tolerances introduce a slight volume discrepancy between the initial (275.4 mm^3^) and recovered (~284.3 mm^3^) states, the shape recovery remains essentially isochoric. Ultimately, such isochoric deformation confirms that the substantial in-plane contraction directly translates into vertical thickening, providing the requisite mass transport for the subsequent localized self-growth.

Crucially, this macroscopic metric functions as a pivotal constitutive parameter for our subsequent microengineering. The experimentally determined shrinkage ratio provides the essential boundary conditions to predict the geometry of laser-induced features. Consequently, these quantified values were employed to quantitatively prescribe the effective shrinkage strain in the FEA discussed in the Section 2.2, ensuring that our simulation-guided rational design possesses high physical fidelity grounded in the material’s intrinsic rheological behavior.

### 2.2. Architecture-Inspired Design and Simulation-Guided Structural Verification

**Figure 2:**
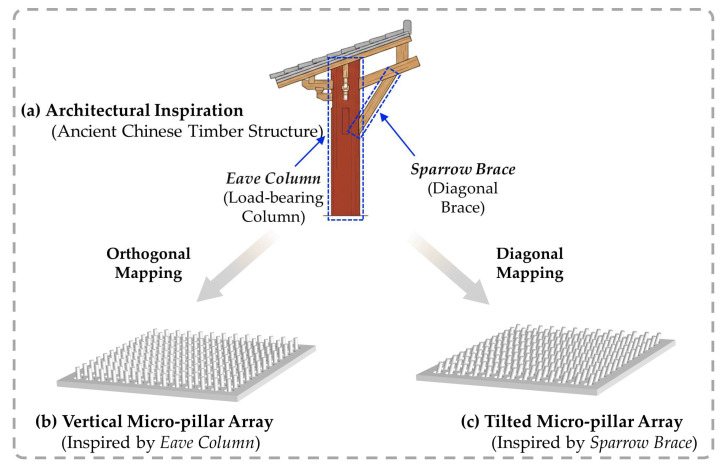
Architecture-inspired structural design: (**a**) Schematic of the ancient Chinese timber structure, identifying the vertical load-bearing *Eave Column* and the diagonally stabilizing *Sparrow Brace* as the architectural prototypes. (**b**,**c**) Structural translation into dielectric micro-architectures, (**b**) vertical micro-pillar arrays resembling the *Eave Column*, and (**c**) inclined micro-pillar arrays originating from the *Sparrow Brace*. This design strategy aims to translate the macro-scale structural resilience of the *Sparrow Brace* into the micro-scale dielectric layer to optimize mechanical responsiveness.

Leveraging the entropy-driven self-growth mechanism quantified in Section 2.1, we developed a structural design strategy to tailor the geometric configuration of the dielectric layer [38,39]. To engineer these microscopic sensing elements, we translated the load-bearing ingenuity of traditional Chinese timber architecture (Figure 2a). This macroscopic framework maintains stability through the structural synergy between the peripheral *Eave Column* for primary vertical support and the *Sparrow Brace* for diagonal pressure distribution [40]. Drawing a structural analogy, we established a conceptual mapping in which the vertical micro-pillar array (Figure 2b) mirrors the orthogonal support of the *Eave Column*, whereas the tilted array (Figure 2c) replicates the diagonal force transfer of the *Sparrow Brace*. Hypothesizing that these macroscopic load-bearing prototypes can be adapted to modulate mechanical stiffness in flexible electronics, we established representative 3D models for the sensor dielectric layer to computationally verify this structural efficacy. To establish a physically grounded baseline for these models, the geometric parameters were derived from the theoretical calculations in Equation (1). For the purpose of theoretical estimation, we defined the effective processing boundary using the nominal laser spot diameter (~20 μm), approximating the interaction volume while neglecting the indeterminate thermal diffusion zone for theoretical clarity. Consequently, based on the principle of volume conservation, a series of distinct geometric models was reconstructed for subsequent computational verification.

**Figure 3:**
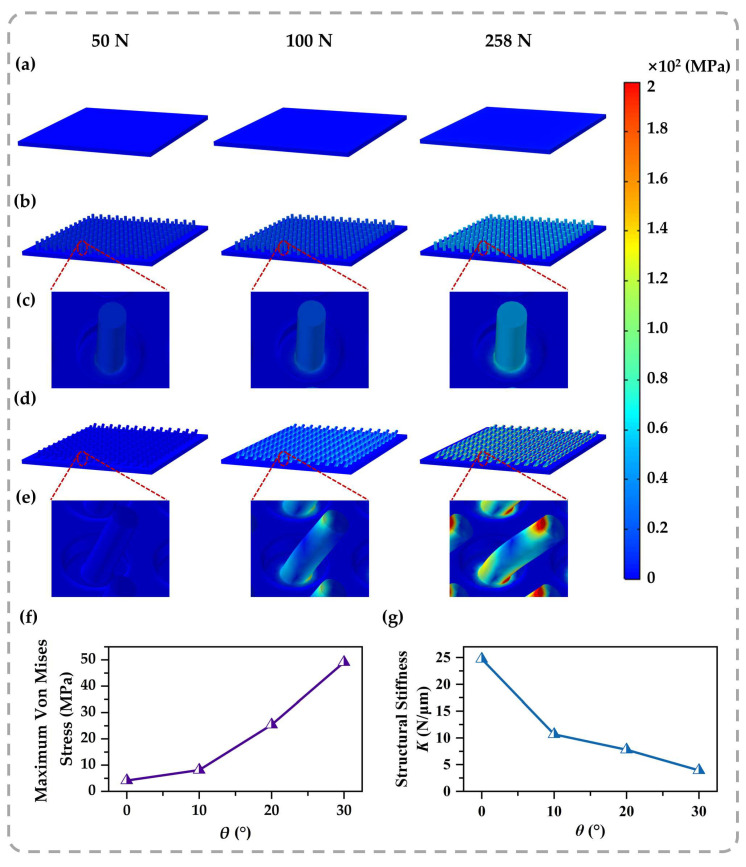
Simulation-guided structural verification and mechanical characterization: (**a**–**e**) Comparative FEA simulations showing the von Mises stress distribution calculated assuming a linear elastic constitutive model under normal loads of 50 N, 100 N, and 258 N. The panels correspond to a planar film reference in (**a**), the vertical micro-pillar array in (**b**,**c**), and the tilted micro-pillar array in (**d**,**e**). Enlarged views in (**c**,**e**) detail the local stress concentration and deformation modes. Unlike the uniform bulk compression of vertical pillars, the tilted geometry drives a bending-dominated response that concentrates intense stress (>200 MPa) at the root, an extreme localization that qualitatively supports the *Sparrow Brace*’s mechanical leverage and amplifies structural sensitivity under external pressures. (**f**) Calculated maximum von Mises stress as a function of the tilt angle *θ*, indicating a transition in the dominant deformation mechanism. (**g**) Structural stiffness *K* versus tilt angle *θ*. This monotonic decrease in stiffness with increasing angle confirms that the tilted architecture effectively lowers mechanical impedance. Parametric studies in (**f**,**g**) were conducted under a constant load of 18.69 N, corresponding to a baseline pressure of 3 MPa for the 90° pillar, to isolate geometric effects.

Comparative FEA under normal loads (50 N, 100 N, and 258 N) elucidates the distinct mechanical behaviors between these designs. Figure 3 shows that the planar film does not respond significantly to stress. Similarly, the von Mises stress in the vertical pillar array (Figure 3b,c) is uniformly distributed along the pillar shaft, which is typical of axial compression [13]. This arrangement replicates the substantial load-bearing stability of the architectural *Eave Column*, with deformation primarily restricted to the vertical axis, yielding significant structural stiffness. It constrains the total deformability and consequently restricts the spatial displacement of the dielectric layer. In sharp contrast, the tilted micro-architecture (Figure 3d,e) breaks this symmetry, demonstrating a distinct bending-dominated behavior resembling a cantilever mechanism. The structural inclination functions as a geometric lever, effectively transforming the vertical normal pressure into a significant bending moment, while stress color mapping in the magnified images indicates a significant stress concentration at the pillar root, theoretically projected to exceed 200 MPa. To clearly visualize this stress transmission, we performed modeling using isotropic linear elastic solids. Thus, these values represent local stress concentrations derived from the linear assumption, serving as theoretical indicators of stress magnitude. This distinct stress distribution contrasts sharply with the uniform compression seen in vertical pillars, suggesting that the external work is efficiently channeled into localized deformation. Consequently, the simulation results demonstrate that the tilted geometry activates a stress-driven mode transformation. In contrast to the bulk compression seen in vertical pillars, this design guides external loads into controllable structural bending, creating a favorable mechanical environment that is theoretically advantageous for sensing response [41]. While this FEA qualitatively demonstrates the trend toward bending-dominated deformation, the idealized isotropic linear elastic model naturally abstracts complex microphysical phenomena. It cannot quantitatively resolve true local stress magnitudes, material nonlinearity, rate dependence, or stress redistribution at the compliant contact interfaces. Consequently, the precise micromechanical mechanisms preventing fracture under these theoretical high stresses fall beyond the quantitative scope of this idealized model. Nevertheless, these theoretical abstractions do not compromise the empirical validity of the sensor’s functionality, which is independently confirmed by the macroscopic performance testing and stability evaluations presented in the next sections.

To quantitatively optimize the geometric parameters, we tested the tilt angle *θ* (0–30° from the *z*-axis) under a strict 18.69 N constant force. This specific load magnitude was calibrated to correspond to a reference stress of 3 MPa in the standard vertical pillar (0°). By maintaining a constant force input, the simulation effectively decouples the geometric influence of the inclination angle from the loading conditions, mimicking real-world tactile scenarios where external force remains constant regardless of sensor deformation. The quantitative results (Figure 3f,g) indicate that increasing the inclination angle significantly attenuates the structural stiffness. Navigating this mechanical trade-off, we deliberately anchored the experimental design at *θ* = 20° to optimally balance structural compliance with physical stability. In addition to optimizing the angle, we assessed the impact of pillar diameters and heights on mechanical performance, as seen in Figure S1 (Supporting Information). The theoretical modeling corroborates that higher aspect ratios favor structural compliance. Preliminary optimizations reveal that excessive aspect ratios inherently threaten both structural robustness and manufacturing consistency. The selected dimensions thus ensure a highly reproducible laser-induced self-growth process, perfectly balancing mechanical performance with fabrication feasibility.

### 2.3. Femtosecond Laser-Enabled Deterministic Self-Growth and High-Precision Fabrication Consistency

**Figure 4:**
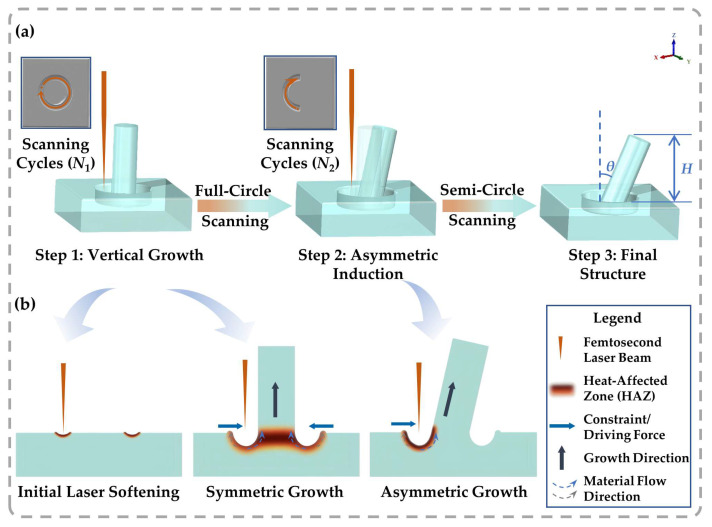
Schematic illustration of the femtosecond laser-induced controllable self-growth strategy based on asymmetric thermal regulation: (**a**) Schematic illustration of the two-step FsLDW strategy for tailoring dielectric layer micro-architectures on BOPS films: Step 1 initiates vertical growth via full-circle scanning (N1 cycles); Step 2 introduces asymmetric-breaking thermal induction via semi-circle scanning (N2 cycles), yielding the final tilted geometry (labeled as Final Structure) with a controllable inclination angle (*θ*). (**b**) Cross-sectional mechanism of the directional tilting. Unlike the isotropic expansion in the vertical growth phase, the asymmetric laser input induces a localized strain mismatch within the heat-affected zone (HAZ). This unbalanced driving force effectively guides the molten material to reconfigure and solidify into a stable inclined structure, analogous to the diagonal load-bearing mechanism of the *Sparrow Brace*.

Guided by the architectural principles and stress-concentration mechanisms established in the Section 2.2, we transitioned from theoretical modeling to experimental realization using a femtosecond laser-induced controllable self-growth strategy [42]. As delineated in Figure 4a, this self-growth protocol creates dielectric layer micro-architectures by leveraging the material’s intrinsic shape memory. Initially, a series of concentric scanning cycles was executed to initiate localized shape recovery, where the intrinsic in-plane shrinkage drives the vertical elongation. The ultrafast energy deposition within femtosecond timescales induces an instantaneous temperature elevation in the focal volume that significantly surpasses the glass transition temperature (*T*_g_) of the BOPS substrate. Crucially, unlike global thermal annealing, which leads to bulk shrinkage, the FsLDW process creates a finely confined HAZ. Within this region, the micropillar formation is governed by the concurrent interplay of partial laser ablation and localized photothermal heating [32]. This specific laser–matter interaction precisely triggers the phase transition and activates the entropy-driven recovery kinetics (as detailed in Section 2.1), facilitating the vertical growth of the polymer phase into a nascent micro-pillar (Step 1). Subsequently, to induce a deterministic structural inclination, a symmetry-breaking thermal field was introduced in Step 2 via asymmetric scanning paths [32,42]. This spatial modulation creates a non-uniform thermal gradient across the pillar base. As elucidated in the cross-sectional mechanism (Figure 4b), this gradient translates into a localized strain mismatch within the HAZ. Unlike the axisymmetric vertical growth in the first phase, the asymmetric energy deposition drives preferential volume recovery on the scanned side. The resulting growth disparity generates a lateral stress differential that functions as an internal “micro-hinge”, effectively pushing the continuously growing pillar to tilt away from the laser-scanning path. The final geometry is thus a synergistic result of localized thermal effect, anisotropic stress release, and rapid solidification, yielding tilted architectures with controllable inclination angles (*θ*).

**Figure 5:**
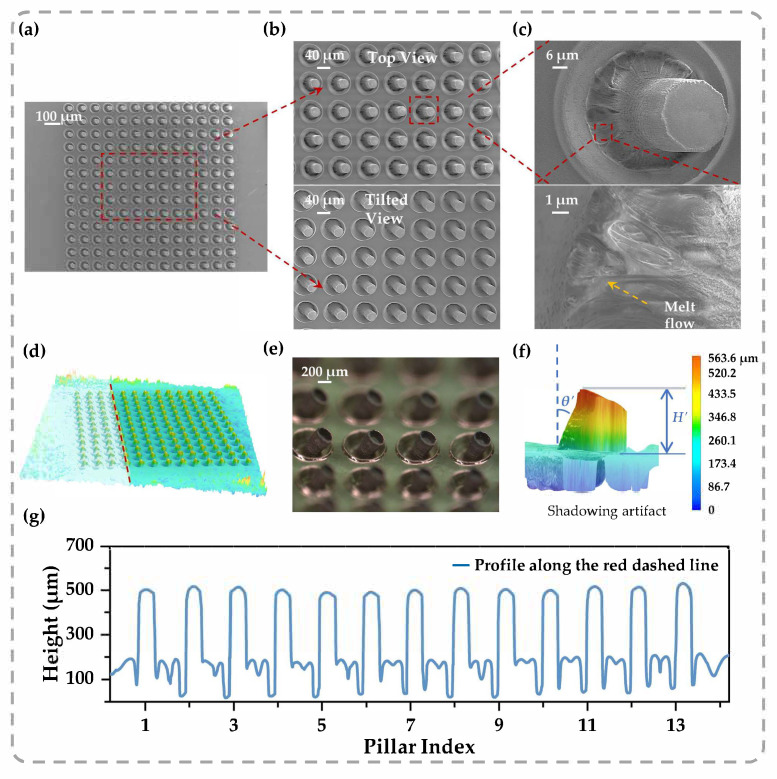
Morphological characterization of the fabricated tilted micro-pillar arrays: (**a–c**) Comprehensive morphological characterization via SEM imaging: (**a**) representative top-view image demonstrating the large-area homogeneity and high periodicity; (**b**) magnified top and tilted views highlighting the structural consistency and quasi-circular top morphology; (**c**) high-resolution surface analysis exhibiting the characteristic layer-by-layer shrinking signatures on the sidewalls, along with melt flow traces at the pillar root and a distinct annular accumulation rim at the periphery. (**d**–**g**) Quantitative 3D topographical analysis via ultra-depth-of-field 3D microscopy: (**d**) 3D pseudo-color reconstruction of the array; (**e**) optical micrograph providing a macroscopic view, visualizing the well-defined tilted cylindrical morphology and the annular rims; (**f**) single-pillar rendering presenting the measured height (*H’*) and tilt angle (*θ’*). Note that the apparent pseudo-trapezoidal profile in the 3D rendering is an optical shadowing artifact induced by the tilted geometry, whereas the actual structural integrity is confirmed by the optical (**e**) and SEM images (**a**–**c**). (**g**) Cross-sectional height profile along the sampling line (Pillar Index 1–13), validating the high reproducibility of the self-growth process.

We performed comprehensive morphological characterizations to assess the geometric precision and spatial uniformity of the FsLDW method. High-resolution SEM (Figure 5a–c) reveals the efficient formation of highly periodic arrays. Closer inspection (Figure 5b) verifies that the individual pillars maintain high structural fidelity across the entire pattern. High-magnification observations (Figure 5c) further uncover the microscopic signatures of the laser–matter interaction, where the periodic textures on the sidewalls attest to the stability of the layer-by-layer modification process, and the distinct traces at the pillar base reveal the localized melt flow dynamics. Specifically, the circular laser scanning trajectory imposes an asymmetric thermal field wherein the internally enclosed region undergoes concentrated inward shrinkage to build the pillar, while the unconfined outer periphery overflows radially to form the distinct annular rim. This peripheral material overflow naturally constrains the volumetric accumulation of the micro-pillar. Despite the intense photothermal interaction, the polymer matrix exhibits no catastrophic fracture, maintaining its viscoelastic nature. This structural integrity ensures the continuous transport of the molten phase from the substrate to the pillar body, yielding a cohesive, monolithic architecture. Complementing the SEM data, ultra-depth-of-field 3D microscopy (Figure 5d–g) provides comprehensive topographical profiling to deliver both macroscopic 3D visualization and rigorous quantitative validation. The cross-sectional profile extracted from the sampling line confirms a remarkably uniform height distribution, firmly establishing the reliability of the FsLDW strategy. Quantitatively, the actual lateral shrinkage ratio (Figure S3), governed by the effective laser processing boundary, aligns well with the macroscopic theoretical values derived from Equation (1). While the measured micro-pillar height is lower than the ideal volume-conservation prediction, this reduction is primarily attributed to inevitable mass loss from oxidative ablation [43] and radial material overflow forming the annular rims (Figure 5c,e). Furthermore, the pillars feature a well-defined inclination angle (*θ’* ≈ 19.4°) that closely approximates the predefined design parameter (*θ* = 20°) (verified in Figure S4), highlighting the extreme precision of the proposed strategy. Collectively, these analyses indicate that the FsLDW method effectively suppresses the stochastic nature of thermal deformation, which is paramount for ensuring uniform pressure distribution and stable signal transduction in subsequent capacitive sensing applications.

### 2.4. Device Integration and Systematic Evaluation of Sensing Performance

**Figure 6:**
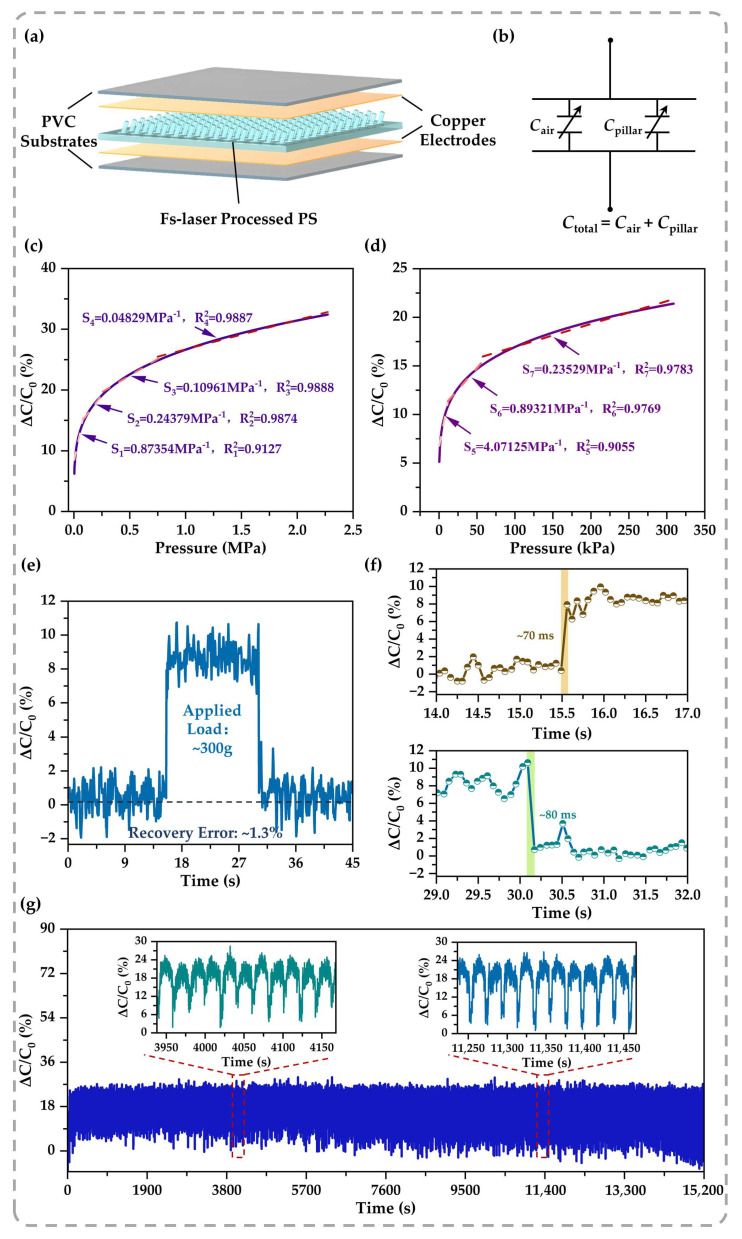
Structural integration and electromechanical sensing performance of the capacitive pressure sensor: (**a**) Exploded view of the sensor assembly, detailing the sandwich configuration of the electrodes and the tilted micro-pillar dielectric layer. (**b**) Equivalent circuit model representing the parallel connection between the micro-pillars (*C*_pillar_) and the air gaps (*C*_air_), which determines the effective capacitance. (**c**,**d**) Static electromechanical sensing capabilities. While the overall response follows a global power-law trend, the reported segment-wise sensitivities are derived from local piecewise linear fits. (**c**) Broad-range sensitivity profile evaluated up to ~2.28 MPa. The global nonlinear trend follows a power-law empirical model, demonstrating robust multi-stage linearity across distinct pressure regimes (S_1_–S_4_). (**d**) Magnified low-pressure sensitivity curve (0–300 kPa). The device easily captures subtle stimuli, which delivers a peak initial sensitivity (S_5_) of ~4.07 MPa^−1^. (**e**) Dynamic response of the sensor under a step load of 300 g. The quasi-rectangular waveform indicates a rapid response and signal stability. A black dashed line represents the initial baseline. Upon unloading, the signal exhibits a negligible recovery error of ~1.3%, which demonstrates the excellent elastic recoverability and low-hysteresis nature of the laser-fabricated micro-architectures. (**f**) Magnified transient profiles extracting the rapid response (~70 ms) and recovery (~80 ms) times derived from (**e**). (**g**) Long-term stability test conducted under a 1 MPa load over a duration of 15,200 s. The insets provide comparative views of 10 consecutive cycles at two representative time intervals, demonstrating the reliable cyclic endurance and structural robustness of the self-grown micro-architectures.

The transition from discrete micro-architectures to a functional sensing device is realized through a conventional sandwich configuration, which incorporates the self-grown tilted micro-pillar array as the dielectric layer (Figure 6a). Electrically, this configuration forms a parallel capacitive system comprising the polymer pillars and interstitial air gaps (Figure 6b). This structural arrangement efficiently converts external mechanical stimuli into measurable capacitance variations, driven by the synergistic modulation of the electrode distance and the effective dielectric constant of the air–polymer composite [44]. Crucially, systematic evaluation of multiple independently fabricated devices (n = 5) confirms that this structural configuration yields highly reproducible electrical properties. Thus, the sensing data discussed below reflect the typical, representative behavior of the sensor batches, with cross-batch variations remaining consistently minimal.

A defining characteristic of the sensor is its remarkably broad sensing range combined with well-defined piecewise linearity. As evaluated in the broad-range sensitivity profile (Figure 6c), the sensor effectively operates up to ~2.28 MPa. Notably, this multi-stage response is exceptionally linear within each distinct pressure regime, maintaining a high correlation coefficient (*R*^2^ > 0.9). This systematic piecewise linearity stems from the progressive, controlled engagement of the tilted micro-architectures with the electrode surface. As pressure increases, the deforming pillars systematically displace the surrounding air, resulting in a stable and predictable capacitance change that significantly simplifies subsequent electronic calibration. To closely examine the sensor’s capability in detecting subtle stimuli, the low-pressure regime (<300 kPa) is magnified in Figure 6d. This detailed profile further resolves the early sensing behavior into distinct linear segments, yielding an initial sensitivity of ~4.07 MPa^−1^ in the first stage. From a structural standpoint, while the macroscopic nominal pressure applied to the sensor is on the order of several MPa, the local stress concentrated at the micro-pillar roots can theoretically exceed hundreds of MPa (as predicted by the FEA in Figure 3). This apparent numerical divergence is a fundamental feature of microstructured interfaces governed by contact mechanics: the macroscopic pressure is calculated over the entire nominal area of the sensor pad, whereas the localized stress is intensely magnified at the confined micro-contact interfaces of the pillar tips. Furthermore, although this highly concentrated theoretical stress exceeds the intrinsic yield strength of the polymer matrix, no catastrophic fracture occurs in the actual device. This structural survivability is primarily attributed to the compliant nature of the sandwich configuration. Under continuous loading, the flexible electrodes and packaging layers undergo coordinated macroscopic deformation, which effectively redistributes the localized load and prevents the isolated mechanical failure of individual micro-pillars.

Beyond the broad sensing range, achieving low hysteresis is critical for dynamic reliability. Step-loading test (Figure 6e) yields a negligible recovery error of ~1.3%. Specifically, this recovery error (*E_r_*) is evaluated using the mean capacitance at stable states, expressed as

(2) $$E_{r}=\frac{{\bar{C}}_{unload}-{\bar{C}}_{initial}}{{\bar{C}}_{load}-{\bar{C}}_{initial}}\times 100\%$$

where ${\bar{C}}_{initial}$, ${\bar{C}}_{load}$, and ${\bar{C}}_{unload}$ denote the mean capacitance before, during, and after the applied load, respectively. This minimal residual signal indicates excellent elastic recovery upon unloading, confirming that the architecture-inspired design effectively accommodates external loads through reversible structural deformation, which fundamentally underpins the low-hysteresis performance. Building on this structural resilience, the sensor clocks agile response and recovery times of ~70 ms and ~80 ms (Figure 6f). Furthermore, this structural resilience extends to long-term cyclic operation. Over a continuous 15,200 s fatigue test under a 1 MPa load (Figure 6g), the sensor maintained its functional integrity throughout the extensive loading period. The testing setup occasionally introduces minor baseline shifts during unloading phases due to limited positional repeatability. Yet, the peak capacitance responses remain remarkably constant. This consistent peak stability confirms the absence of macroscopic structural fatigue, highlighting the long-term durability of the self-grown micro-architectures. While the continuous and rapid loading–unloading profile of this fatigue test precludes the extraction of cycle-by-cycle quasi-static hysteresis loops, the sustained macroscopic stability effectively validates the sensor’s structural reversibility.

Beyond sustained mechanical endurance, the intrinsic thermal stability of the device is equally paramount for real-world applications. While the localized self-growth process is driven by photothermal heating above the BOPS film’s glass transition temperature (*T*_g_) of approximately 100 °C [45], the anticipated operating environments for the flexible sensor are typically confined to ambient conditions. Because these practical working temperatures remain substantially below this critical thermodynamic threshold, the fabricated micro-architectures inherently preserve their structural integrity, effectively precluding any risk of undesired heat-induced deformation. Furthermore, as illustrated in Table 1, the present work distinguishes itself from recent representative studies through the competitive sensing performance enabled by our material and fabrication strategy. Ultimately, these results underscore the efficacy of the femtosecond laser-induced self-growth strategy. By guaranteeing the deterministic creation of complex micro-architectures, this approach opens a highly practical route for designing high-performance flexible sensors.

## 3. Experimental Section

### 3.1. Preparation of Dielectric Layers

Commercially available BOPS films (thickness: 0.3 mm, Guangzhou Aishangji Technology Co., Ltd., Guangzhou, China) were sectioned into 20 × 20 mm substrates. Micro-architectures were patterned onto the BOPS surface using a programmed femtosecond laser system (FemtoYL-40, Wuhan Anyang Laser Technology Co., Ltd., Wuhan, China) operating at a center wavelength of 1030 nm, a pulse duration of 300 fs, and a repetition rate of 25 kHz. The laser power was fixed at 0.399 W with a focal spot diameter of ~20 μm. Utilizing Ezcad2.14.9 software for precise spatial manipulation, the beam was translated along pre-designed 2D trajectories consisting of overlapping circular and semi-circular paths with a diameter of 0.467 mm. These structural units were arrayed in a checkerboard pattern with a center-to-center spacing of 0.767 mm, ultimately yielding a 13 × 13 matrix of tilted micro-pillars over a 10 × 10 mm effective area. The circular and semi-circular patterns were fabricated at a constant scanning speed of 40 mm/s, employing 45 (N1) and 30 (N2) scanning repetitions, respectively.

### 3.2. Sensor Assembly and Integration

The capacitive pressure sensor was assembled using a conventional sandwich configuration (Figure 4a). Polyvinyl chloride (PVC) substrates (30 × 30 mm) were utilized for structural encapsulation. Double-sided conductive copper foil tapes with a thickness of 0.05 mm were attached to the PVC substrates to serve as functional electrodes. To facilitate external electrical connections, the copper foil was extended 30 mm beyond the substrate edge. Device integration was finalized by directly laminating the as-prepared dielectric layer between the top and bottom electrode assemblies.

### 3.3. Characterization and Testing

Surface morphologies of the micro-architectures were examined using a scanning electron microscope (SEM, S-3400N, Hitachi Ltd., Tokyo, Japan) following standard gold sputtering (Figure 5a–c). Three-dimensional geometric profiling was conducted via an ultra-depth-of-field digital microscope (VHX-7000, Keyence Co., Ltd., Shanghai, China) (Figure 5d–g). For electromechanical characterization, dynamic compressive loads were applied using a universal testing machine (UTM 2502, Shenzhen Suns Technology Stock Co., Ltd., Shenzhen, China), with the corresponding capacitance responses simultaneously recorded by an LCR meter (TH2832, Changzhou Tonghui Electronics Co., Ltd., Changzhou, China). All capacitance measurements were standardized at an AC voltage of 2 V and a testing frequency of 1 kHz.

## 4. Conclusions

This work presents a conceptually novel flexible capacitive pressure sensor that resolves the persistent trade-off between wide detection ranges and low mechanical hysteresis. By exploiting the highly localized thermal manipulation of programmable FsLDW and the entropy-driven shape memory of BOPS films, we achieved the mask-free, in situ fabrication of architecture-inspired *Sparrow Brace* microarrays. The precise structural geometry (height: 346.6 μm, inclination: 19.4°) aligns seamlessly with our data-driven FEA models. Crucially, these simulations show that this tilted architecture translates the dominant deformation mode from conventional bulk compression to structural bending. This physical mechanism suppresses the effects of polymer viscoelastic creep under the tested conditions, endowing the sensor with an exceptionally broad working range (up to ~2.28 MPa) and a negligible recovery error (~1.3%). These metrics are complemented by an agile dynamic response (~70/80 ms), high linear predictability (*R*^2^ > 0.9), and consistent cyclic stability over 15,200 s. Rather than relying on complex material modifications to chase peak sensitivity, this structural design prioritizes reliable load-bearing capacity and highly reversible signal responses. Bridging macroscopic architectural concepts with micro-device engineering, this work offers a generalized physical strategy to reduce the contribution of bulk-compression-dominated viscoelastic effects under the tested conditions, effectively alleviating the inherent viscoelastic bottlenecks of soft materials. Beyond the BOPS dielectric layer investigated here, this fs-laser-induced self-growth strategy exhibits excellent generality as a micro-manufacturing method. It is theoretically applicable to a broad range of other prestrained thermoplastic shape-memory polymers, enabling the versatile selection of sensing materials. Ultimately, this approach holds tremendous potential for advanced applications in human–machine interfaces and intelligent robotic perception.

## Figures and Tables

**Table 1:** Comparison of sensing performance with recent representative soft sensors.

Architecture	Fabrication Method	Working Range	Hysteresis
Dome-shaped [14] (Bulk compression)	Template	0–200 kPa	Not reported
Tilted pillars [21] (Structural bending)	Template	0–30 kPa	3.46%
Lantern-inspired [46] (Bulk compression)	3D Printing	0–100 kPa	2.05%
This work (Structural bending)	FsLDW	0–2.28 MPa	~1.3%

## Data Availability

The original contributions presented in this study are included in the article. Further inquiries can be directed to the corresponding author.

## Supplementary Materials

The following supporting information can be downloaded at: https://www.mdpi.com/article/10.3390/s26092784/s1, Figure S1: Parametric study of geometric factors; Figure S2: Quantitative geometrical characterization of the fabricated tilted micro-pillar; Figure S3: Geometric characterization of the micro-pillar array; Figure S4: Vertical topographical analysis of the micro-pillar array via ultra-depth-of-field microscopy.
