# Supplementary material for: Wide-Range, Low-Hysteresis Soft Sensor with Architecture-Inspired Design Enabled by Femtosecond Laser-Induced Self-Growth
Source: Sensors (Basel). 2026 Apr 29;26(9):2784. doi: 10.3390/s26092784 (PMC13165758; doi:10.3390/s26092784)
Supplement: Supplementary file 1 [file sensors-26-02784-s001.zip › sensors-4248253-supplementary.pdf]

sensors

MDPI

Article

Wide-Range, Low-Hysteresis Soft Sensor with Architecture-Inspired Design Enabled by Femtosecond Laser-Induced Self-Growth

Ziyue Yu [1,2], Changhao Ji [1,2], Xinyue Gao [1,2], Yu Li [3], Cheng Yang [1,2], Fawei Guo [1,2], Jianglin Fu [1,2], Yin Feng [1,2], Hongxuan Zhao [1,2] and Yu Long [1,2,*]

[1] State Key Laboratory of Featured Metal Materials and Life-cycle Safety for Composite Structures, Guangxi University, Nanning 530004, China; 18240438586@163.com (Z.Y.); 2211401014@st.gxu.edu.cn (C.J.); gao_xy1123@163.com (X.G.); 2211401010@st.gxu.edu.cn (C.Y.); 2211392007@st.gxu.edu.cn (F.G.); fjlbee@st.gxu.edu.cn (J.F.); fengyinzi06@163.com (Y.F.); 2411301074@st.gxu.edu.cn (H.Z.)

[2] Institute of Laser Intelligent Manufacturing and Precision Processing, School of Mechanical Engineering, Guangxi University, Nanning 530004, China

[3] School of Mechanical Engineering, Beijing Institute of Technology, Beijing 100089, China; 3120255446@bit.edu.cn

* Correspondence: longyu@gxu.edu.cnSupporting Information Includes:

One PDF file containing supplementary finite element analysis of geometric parameters, high-resolution optical microscopy images and raw geometric measurement data of the fabricated micro-pillar arrays, presented as Supporting Figures S1-S4.

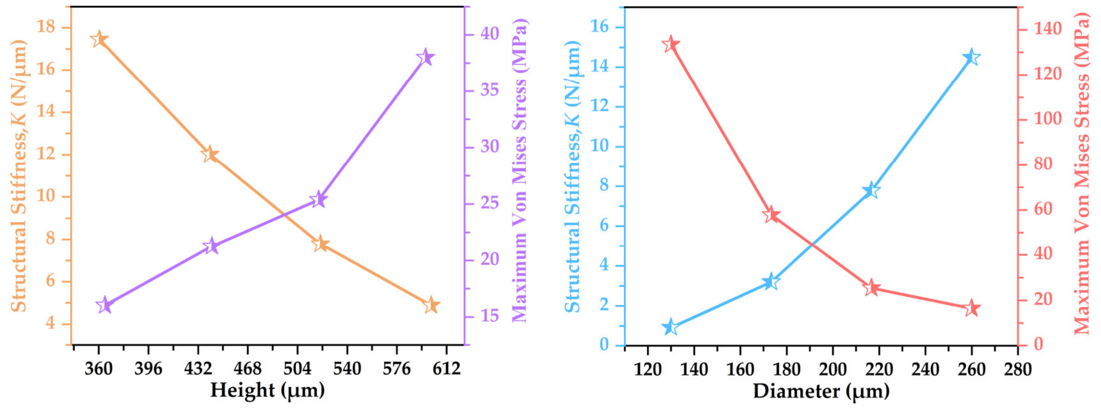

Figure S1. Parametric study of geometric factors. (a) Evolution of stiffness and stress with diameter. **(b)** Influence of height on mechanical response. The analysis highlights the compliance benefits of higher aspect ratios. Consequently, the experimental geometry was tailored to maximize compliance within the stable processing window of the laser-induced self-growth method.

 https://doi.org/10.3390/s26092784

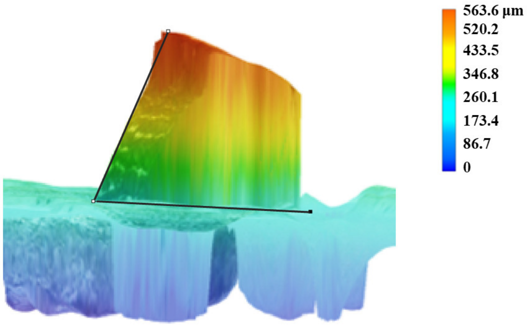

	Area	Mean	Min	Max	Angle
1	0	0	0	0	70.555

Figure S2. Quantitative geometrical characterization of the fabricated tilted micro-pillar. (Top) 3D topographical reconstruction of a single self-grown micro-pillar obtained via Ultra-Depth-of-Field Microscopy. **(Bottom)** Measured data table revealing an elevation angle of ~70.6° relative to the substrate. This corresponds to a vertical inclination angle (θ') of ~19.4°, which aligns exceptionally well with the predefined design parameter (θ= 20°) established in the FEA simulation, validating the high geometric fidelity of the FsLDW strategy.

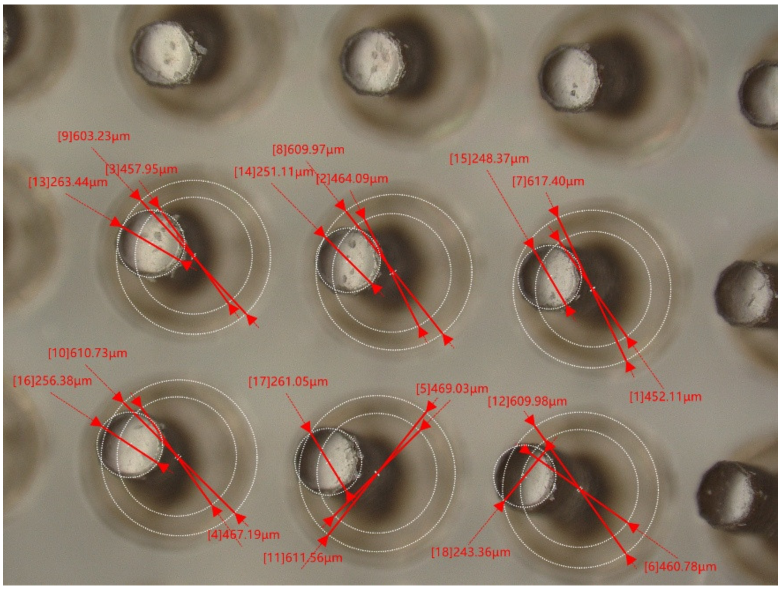

Figure S3. Geometric characterization of the micro-pillar array. The annotated features delineate the effective thermal boundary (D_1, outer), the laser scanning trajectory (D_2, middle), and the projected pillar diameter (D_3, inner). Through statistical analysis, the average dimensions were calculated to be $D_1 \approx$ 610.48 μm, $D_2 \approx$ 461.86 μm, and $D_3 \approx$ 253.95 μm. Notably, the measured scanning path (D_2) closely matches the 467 μm digital parameter settings detailed in Section 3.1. To determine the physical shrinkage behavior, the projected diameter (D_3) was geometrically corrected via the cosine of the inclination angle (θ'), yielding an actual pillar diameter of ~269.31 μm. The resulting lateral shrinkage ratio is calculated to be 55.89%, which is highly consistent with the macroscopic shrinkage characteristics derived in Section 2.1. Collectively, these quantitative consistencies demonstrate the uniformity of material properties and the stability of the fabrication process.

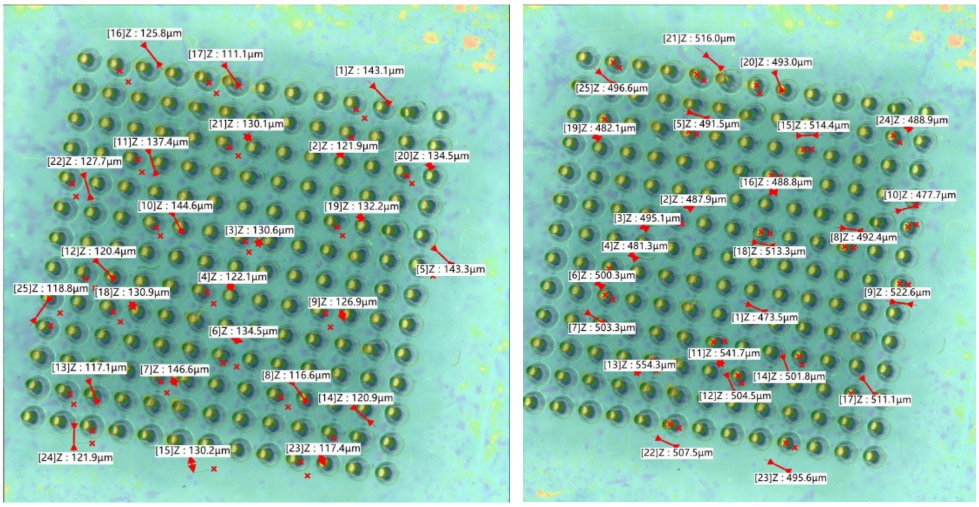

Figure S4. Vertical topographical analysis of the micro-pillar array via ultra-depth-of-field microscopy. The quantitative measurements characterize two critical vertical features: **(Left)** the depth of the surrounding grooves, and **(Right)** the total vertical span measured from the groove bottom to the pillar peak. Statistical analysis yields an average groove depth of ~128.26 µm and a total vertical span of ~501.41 µm. To compare with the theoretical volume-conservation model, the measured total span was geometrically converted to an actual pillar length of ~484.19 µm. The substantial difference between this empirical value and the theoretical prediction (~659.09 µm) arises from material ablation and the formation of outer rims, indicating a deviation from ideal volume conservation.

https://doi.org/10.3390/s26092784
